# Reprogramming miRNAs global expression orchestrates development of drug resistance in BRAF mutated melanoma

**DOI:** 10.1038/s41418-018-0205-5

**Published:** 2018-09-25

**Authors:** Luigi Fattore, Ciro Francesco Ruggiero, Maria Elena Pisanu, Domenico Liguoro, Andrea Cerri, Susan Costantini, Francesca Capone, Mario Acunzo, Giulia Romano, Giovanni Nigita, Domenico Mallardo, Concetta Ragone, Maria Vincenza Carriero, Alfredo Budillon, Gerardo Botti, Paolo Antonio Ascierto, Rita Mancini, Gennaro Ciliberto

**Affiliations:** 1 IRCCS, Regina Elena National Cancer Institute, Rome, Italy; 2 Department of Experimental and Clinical Medicine, University “Magna Graecia” of Catanzaro, Catanzaro, Italy; 3 Department of Molecular and Clinical Medicine, University of Roma “Sapienza”, Rome, Italy; 4 IRCCS, National Cancer Institute of Naples “Fondazione G. Pascale”, Naples, Italy; 5 Division of Pulmonary Disease and Critical Care Medicine, Virginia Commonwealth University School of Medicine, Richmond, VA USA; 6 Department of Molecular Virology, Immunology, and Medical Genetics, The Ohio State University Comprehensive Cancer Center, Columbus, OH USA

**Keywords:** Tumour biomarkers, Oncogenes

## Abstract

Drug resistance imposes severe limitations to the efficacy of targeted therapy in BRAF-mutated metastatic melanoma. Although this issue has been mitigated by the development of combination therapies with BRAF plus MEK inhibitors, drug resistance inevitably occurs with time and results in clinical recurrences and untreatable disease. Hence, there is strong need of developing new combination therapies and non-invasive diagnostics for the early identification of drug-resistant patients. We report here that the development of drug resistance to BRAFi is dominated by a dynamic deregulation of a large population of miRNAs, leading to the alteration of cell intrinsic proliferation and survival pathways, as well as of proinflammatory and proangiogenic cues, where a prominent role is played by the miR-199b-5p/VEGF axis. Significant alterations of miRNA expression levels are detectable in tumor biopsies and plasma from patients after disease recurrence. Targeting these alterations blunts the development of drug resistance.

## Introduction

The natural history of metastatic melanoma has recently changed, thanks to the development of novel immunotherapies and targeted therapies, which have significantly improved patients’ survival [1, 2]. Kinase inhibitors (KIs) of the MAPK pathway were developed following the discovery that BRAF mutations occurring in nearly 50% of patients are major oncogenic drivers of melanoma proliferation and survival [2, 3]. This evidence led to the clinical development initially of BRAF inhibitor monotherapy, subsequently of combo therapies with BRAF and MEK inhibitors because resistance to BRAF inhibitors is often characterized by bypass mutational or non-mutational events leading to reactivation of MAPK signaling [4, 5]. Unfortunately, although BRAF/MEK combo-therapy leads to a more durable control of tumor growth and to prolonged survival, it is unable to completely eradicate disease. Acquired resistance manifests itself with tumor recurrences growing often *in situ* from residual tumor cells after initial shrinkages [6]. This scenario is further complicated by the evidence that approximately 10–15% of melanoma patients harboring BRAF-mutations do not respond *ab initio* to first line therapy, and that 40–50% of patients show only partial responses [7]. Hence both intrinsic and acquired resistance are major hurdles to effective and durable therapy.

During last years, several studies directed to understand the molecular basis of resistance to KIs have identified genetic and phenotypic mechanisms [8]. Interestingly, the same genetic alterations have been identified both in BRAFi mono-therapy resistant as well as in BRAFi + MEKi double-resistant tumors and mostly converge in the reactivation of MAPK signaling [6, 8]. In contrast, phenotypic mechanisms are linked to the activation of a highly heterogeneous and dynamic set of adaptive responses fueled by tumor cell plasticity [9–11]. The preponderance of non-genomic changes occurring in melanoma with acquired resistance to MAPK inhibitors suggest that dynamic alterations take place early on after drug exposure [12]. It has been observed that clinical objective responses occur in parallel with the development of tolerance mechanisms linked to transcriptome reprogramming. These transcriptome changes involve both cell intrinsic and cell extrinsic mechanisms, and reset the interaction of melanoma cells with cells of the tumor microenvironment [13].

Inspired by the evidences that several transcriptional and epigenetic events are at play to orchestrate the development of KIs resistance in melanoma, we started to focus on the major class of non-coding RNAs, i.e., microRNAs. These short regulatory RNAs of 19–24 long nucleotides are widely known to play a key role virtually in every kind of human cancer and also in the development of resistance to different anti-neoplastic therapies [14]. In this context we started to assess deregulation of miRNA expression as a major non-genomic alteration at the basis of *de novo* drug resistance in melanoma. This led to the identification of patterns of miRNAs which impact on the development of melanoma and of downstream pathways controlled by them [14, 15]. These initial findings were further corroborated by the recent discovery of the novel miR-579–3p as a regulator of melanoma development and drug resistance [16]. This oncosuppressor miRNA controls the expression of two oncoproteins BRAF and MDM2, is strongly downregulated in BRAF-mutated melanomas and is able to impair the development of resistance to MAPK inhibitors.

However, the great heterogeneity of human melanoma samples strongly suggests the necessity to conceive a more comprehensive approach to the study of miRNA involvement in drug resistance. Hence, in this work we have performed a large study of the entire miRNAome changes during the progressive development of drug resistance in vitro. This has allowed to identify a larger population of miRNAs deregulated in this process and to delineate the wide set of modulated intracellular pathways that affect both cell intrinsic cell growth behaviors, as well as melanoma cell interactions with the tumor microenvironment such as cell migration and angiogenesis. Here we provide strong evidence that simultaneously interfering with the expression of a subset of the identified miRNAs is able to block or revert development of drug resistance and that detection of miRNAs disregulation correlates with drug response in tumor samples from patients treated with inhibitors of the MAPK pathway. These findings together have important therapeutic and diagnostic implications.

## Materials and methods

### Cell lines

All cell lines were routinely tested for Mycoplasma, and cell line and subline identities have been ensured by RNA-seq at routine intervals during the course of this study for banking and experimental studies. All sensitive and BRAFi-resistant human melanoma cell lines used in this work have been previously described [16]. A375^DR^ cells were selected in the presence of both BRAF and MEK inhibitors. BRAF-V600 mutations were evaluated through Sanger method from genomic DNA extracted from M14 and WM266 melanoma cells. Human umbilical vein endothelial cells (HUVEC)s were grown as described in the work by Bifulco et al [17].

### Antibodies, western blot and reagents

Antibodies against GAPDH, Bcl-2 and VEGF were obtained from Santa Cruz Biotechnology. Vemurafenib and trametinib were obtained from Selleck Chemicals. TaqMan probes for GAPDH, VEGFA, TIMP2, PTPN14, HIF-1α, Bcl-2, CCL3, IL1b, CCL5, tgfB, CXCL8, CSF3, PDGFb, CCL20, miR-4443, miR-4488, miR-204-5p, miR-199b-5p, miR-630, miR-1234, ts-3676, miR-145-5p and RNU48 were purchased from Applied Biosystems. Primers relative to H3, RAD51, Notch1 and FOXM1 have been used in the work by Canu et al [18]. Western Blot analysis were performed as previously described [11, 16].

### RNA extraction and real-time pcr analysis

RNA was extracted using TRIzol method (Invitrogen) and quantitated by spectrophotometry. Real-time PCR was assayed by TaqMan Gene Expression Assays (Applied Biosystems).

### Nanostring analysis

To perform Nanostring analysis 100 ng of total RNA were hybridized to the array in the nCounter miRNA Expression Assay v1 (NanoString Technologies, Seattle, WA, USA) following the manufacturer’s instructions. This technology allows direct and digital counting of 800 human miRNAs without amplification reactions. Bioinformatic analysis consider the significantly up- or downregulated miRNAs with at least two-fold changes as compared to controls.

### Target genes prediction of miRNAs and pathway analysis

We performed predictions of miRNA complementarity to 3′-untranslated regions (UTRs) in mRNAs using three commonly used tools for target prediction: TargetScanHuman 6.2 (http://www.targetscan.org/), PITA, and Miranda (http://www.microrna.org/) as previously done [14, 15].

### Cytokinome evaluation

Levels of 27 cytokines, chemokines, and growth factors were evaluated at the same time by the multiplex biometric ELISA-based immunoassay, according to the manufacturer’s instructions (Bio-Plex Bio-Rad). For the complete list see Fig. 5a. Protein levels were quantified using a Bio-Plex array reader (Luminex, Austin, TX, USA) and a standard curve. A fold change greater than 1.3 was considered significant by evaluating the ratio between the cytokine levels in resistant cells compared to those wild type.

### ROC curves

Receiver operating characteristic (ROC) curves were plotted to estimate the predictive value of four miRNAs, to compute optimal cutoffs for any given feature, to generate performance tables for sensitivity, specificity, and confidence intervals at different cutoffs and to select combinations of features to create biomarker models.

### Cell proliferation assay and in vitro colony formation assay

Viability of cells was examined with 3-(4,5-dimethylthiazol-2-yl)−2,5-diphenyltetrazolium bromide Cell Titer 96 AQueous One Solution Cell Proliferation Assay (Promega), according to the manufacturer’s protocol. The plates were analyzed in a Multilabel Counter (Bio-Rad Laboratories). Cells viability was also determined by crystal violet staining. Briefly, the cells were stained for 20 min at room temperature with staining solution (0, 5% crystal violet in 30% methanol), washed four times with water and then dried. Cells were then dissolved in a Methanol/SDS solution and the adsorbance (595 nm) was read using a microplate ELISA reader.

### Human samples

Total RNA was extracted from the FFPE samples from 14 matched tumors from patients before and after the development of resistance to MAPKi, as previously done [16]. Circulating miRNA were extracted from the plasma of 25 melanoma patients before the beginning of therapy and at disease progression through miRNeasy Mini Kit (Qiagen) according to the manufacturer’s instructions. Real-time PCR was assayed as described above. The use of human samples was approved by Istituto Pascale’s Ethical Committee with the protocol DSC/2893 on April 11, 2015. All patients signed a general informed consent, which allowed use of this material for research purposes and which was analyzed in an anonymous manner at the Istituto Nazionale per la Cura dei Tumori “Fondazione G. Pascale".

### Statistical and clinical data analysis

Data from at least three separate experiments are presented as means ± SD. P values were calculated using Student’s t test, except for the ones that involve circulating miRNAs, which were also calculated with Wilcoxon matched pairs test. Significance levels has been defined as *P* < 0.05. All experiments shown, except for the ones that involve clinical samples, were performed independently at least three times. Heatmap was evaluated to correlate the expression values of four miRNAs between them by Pearson correlation coefficients. Data of circulating miRNAs were normalized using two different methods: global mean normalization (GMN) and NormFinder model [19]. TCGA Skin Cutaneous Melanoma data (*n* = 501) relative to miR-204-5p and miR-199b-5p expression levels were obtained querying the free online database PROGmiR [20].

### Cell migration and tube formation assays

Cell migration was monitored in real time using the xCELLigence Real Time Cell Analysis (RTCA) technology (Acea Bioscience) [21]. Lower chambers were filled with serum-free medium (CTRL) or undiluted conditioned media from wild type WM266 or resistant WM266 (WM266^R^) cells. WM266 cells (2x104 cells/well) were seeded on filters in serum-free medium. Cell migration was monitored for 12 h, and each experiment was performed at least twice in quadruplicate. Slope represents the change rate of cell index values generated in a 0–6 h time frame. Tube formation assays on WM266 or their resistant counterparts cells were performed as previously described in the work by Bifulco et al. [17]. To quantify tube formation, images were acquired and the number of tubes formed by cord-like structures exceeding 100 μm in length were visualized using Axiovision 4.8 software (Carl Zeiss) and counted [17].

## Results

### Significant changes in whole miRNAome expression characterize evolution of drug resistance to BRAF inhibitors in human melanoma

**Fig. 1:**
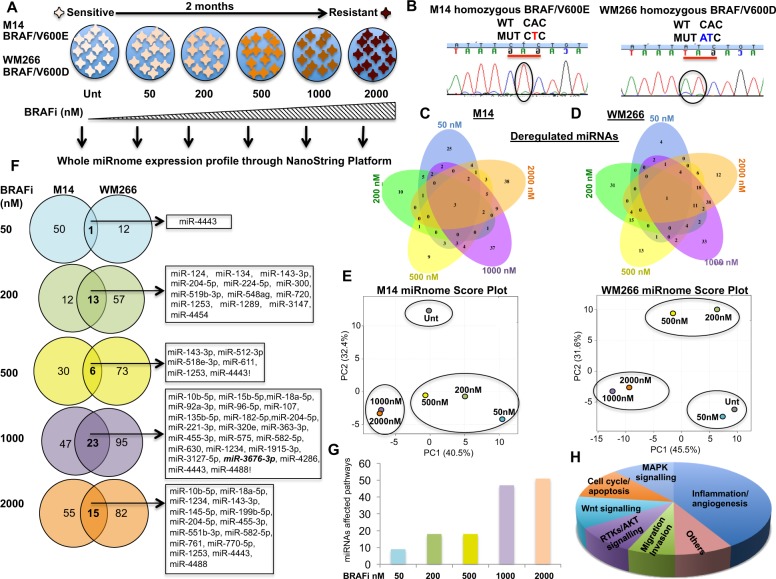
Changes in the expression of the entire miRNAome characterize evolution of BRAFi-resistance in human melanoma cells. **a** Schematic representation of Nanostring study in which M14 and WM266 melanoma cells selected for two months with increasing doses of a BRAF inhibitor are subjected to whole miRNAome profiling. **b** Genomic DNA from M14 and WM266 evaluated by Sanger method confirming BRAF V600 mutational state. **c**, **d** Venn Diagrams showing that each selection step is characterized by a distinct set of miRNAs expression changes in M14 (**c**) and WM266 (**d**) cells. E, Principal Component Analysis confirming that changes of the entire miRNome expression (*n* = 800 miRNAs) characterizing different drug sensitivity states. **f** Venn Diagrams showing the common deregulated miRNAs at each step of the selection. **g** The number of pathways affected by commonly deregulated miRNAs increasing with stepwise drug exposures. **h** Bioinformatics analysis revealing the most relevant intracellular pathways affected by deregulated miRNAs

Very little is known about the changes affecting the entire miRNAome during the progressive development of drug resistance to MAPK inhibitors. In order to address this question we followed the “road to resistance” approach depicted in Fig. 1. Two human BRAF-mutated cell lines bearing different mutations (M14/V600E; WM266/V600D) were exposed to increasing drug concentrations (from 50 nM to 2 μM every two weeks for a total period of 2 months) (Fig. 1a). BRAF mutational status in these two melanoma cell lines was confirmed by direct sequencing (Fig. 1b). At each stepwise drug increase total RNA was extracted and subjected to miRNAome profiling using the Nanostring platform (nCounter Human v1) (Fig. 1a). This technology allows direct and digital counting of 800 human miRNAs without amplification reactions. Analysis of the results allowed the identification of miRNAs significantly up- or downregulated with at least two-fold changes as compared to controls. The results, depicted as Venn Diagrams in Fig. 1c and d, show that each selection step is characterized by a distinct set of miRNAs expression changes, with a shared set of miRNAs deregulated between the different selection steps (for complete statistical analysis see Supplementary Table S1A and B). The most relevant finding was the progressive deregulation (up or down) of a growing number of miRNAs during the selection process. At the highest drug concentrations (i.e. 1 μM and 2 μM BRAFi), 118 and 97 miRNAs ( > 14% and > 12% of total miRNAs analyzed) in WM266 and 70 and 68 miRNAs (approaching 8% of miRNAs analyzed) in M14 were deregulated as compared to the respective sensitive cells (the entire list of statistically significant deregulated miRNAs is reported in the Supplementary Table S1C). This finding underscores a major rewiring of the entire miRNome population in drug resistant vs. sensitive cells.

To confirm this finding, Principal Component Analysis (PCA) [15] of Nanostring data was carried out. The results (Fig. 1e, every dot represents cell populations at a given drug dose), confirmed that changes of the entire miRNome expression (*n* = 800 miRNAs) are able to distinguish different drug sensitivity states. In particular, it is possible to separate drug sensitive cells (untreated or untreated plus 50 nM depending upon cell line) vs. mildly resistant (200–500 nM drug) vs. strongly resistant (1–2 μM). The highest steps of selection (i.e. 1 μM and 2 μM BRAFi) correspond to the greatest number of commonly deregulated miRNAs between the two cell lines (Fig. 1f and Supplementary Fig. S1A). In contrast, the lower steps of selection (i.e., 50–200–500 nM) shared only a small number of deregulated miRNAs with the highest selection steps (Supplementary Fig. S1B), thus suggesting a dynamic switch between a low to high resistance state. In summary the miRNAome of drug resistant cells is significantly different from that of drug sensitive cells and it is possible to distinguish different drug sensitivity states by measuring miRNA expression levels.

We then performed an analysis of the predicted molecular targets of the commonly deregulated miRNAs. To this purpose, we used three available prediction algorithms, TargetScanHuman 6.2, PITA and Miranda and considered only target genes predicted by at least two out of the three algorythms. The resulting gene list was used for a functional annotation analysis of pathways [14, 15] Of notice, the number of pathways affected by commonly deregulated miRNAs between the two cell lines is relatively low at low drug concentrations up to 500 nM, but dramatically increases at the highest drug exposures (Fig. 1g). In particular, 148 and 176 were the predicted mRNA targets of commonly deregulated miRNAs between the two cell lines at the doses of 1 and 2 μM, respectively (for the complete list see Supplementary Fig. S1D and Supplementary Table S1D). Coherently with this a high proportion of predicted target mRNAs were shared between the 1 and 2 μM steps of selection (approaching 70% of the total) (Supplementary Fig. S1C). Among them, there were both known oncogenes such as *BCL2*, *PDGFB* and *KRAS* targeted by downregulated miRNAs and oncosuppressors, such as MAPK13, NCOR2 and BAX, targeted by upregulated miRNAs (Supplementary Table S1D). Importantly, besides intracellular pathways responsible for cell intrinsic growth deregulation such as MAPK, AKT, Wnt signaling and cell cycle/apoptosis, a prominent involvement was observed for pathways responsible for cancer cell extrinsic deregulation of pro-angiogenic and proinflammatory cues (Fig. 1h). These findings are of great interest since perturbation of the tumor microenvironment constitutes a hallmark of melanoma drug resistance [15, 22].

Next we decided to validate the Nanostring data by Real-Time-PCR on a subset of deregulated miRNAs. To this purpose we used a total of four matched BRAF sensitive vs. drug-resistant cell line; namely besides the initial M14 and WM266 cells, also LOX IMVI and A375 (both BRAF-V600E). Again, we selected resistant cells for two months in the presence of increasing concentrations of a BRAFi and stored RNAs at each step. Four miRNAs were chosen: two upregulated (miR-4443 and miR-4488, called also UPMIRNAs) and two downregulated (miR-204-5p and miR-199b-5p, called also DOWNMIRNAs), because deregulated in common between M14 and WM266 at the highest drug concentrations (Fig. 1f). Results (Supplementary Fig. S2A) confirmed that miR-4443 and miR-4488 were strongly increased in all four BRAFi-resistant melanoma cell lines tested, whereas miR-204-5p and miR-199b-5p were significantly downregulated in the same conditions. In addition, the deregulation of these miRNAs was also confirmed in a cell line rendered double resistant to both BRAF and MEK inhibitors (called A375^DR^) (Supplementary Fig. S2B). These data taken together suggest that miR-4443 and miR-4488 could act as facilitators of melanoma drug resistance, while miR-204-5p and miR-199b-5p could antagonize drug resistance.

Furthermore, we were also able to validate Nanostring data of other four additional miRNAs (Supplementary Fig. S2C). Hence, in summary, qRT-PCR data showed excellent agreement with Nanostring analysis, validating the magnitude and the directionality observed for the deregulation of most miRNAs. Interestingly, among them, we also identified and validated miR-3676-3p, originally described as a miRNA, but recently assessed to belong to a new class of small noncoding RNAs, tRNA-derived small RNAs (tsRNAs) which have been suggested to exert an oncogenic or oncosuppressive role in human tumors [23].

### Shared miRNAs deregulated in drug-resistant melanoma cells potently affect cell growth by acting on cell intrinsic pathways

**Fig. 2:**
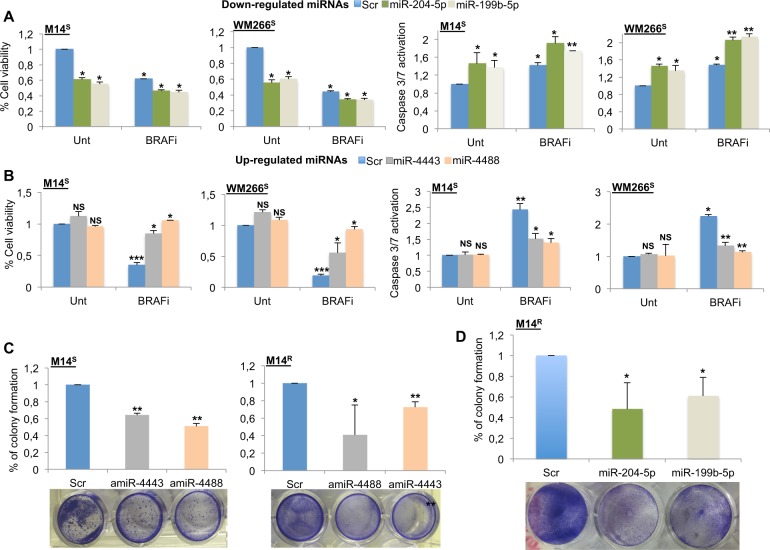
Shared deregulated miRNAs of drug-resistant melanoma cells affect proliferation and apoptosis in the presence or not of a BRAFi. **a**, **b** Effects of miR-204-5p, miR-199b-5p (**a**), miR-4443 and miR-4488 (**b**) transient overexpression on cell viability (left panels) and on Caspase 3/7 activation (right panels) in the presence or not of a BRAFi in M14 and WM266 cells. **c** Specific silencing of miR-4443 and miR-4488 affecting M14S and M14R cell colony formation. **d** Transient enforced expression of miR-204-5p and miR-199b-5p reducing M14R cell colony formation

Next, we assessed the biological consequences on melanoma cell proliferation and apoptosis induction of overexpressing or downregulating the four selected miRNAs above, by transient transfections in drug sensitive M14 and WM266 cells in the presence or not of a BRAFi. Enforced expression of the two downregulated miRNAs (DOWNMIRNAs) miR-204-5p and miR-199b-5p, not only inhibits *per se* cell proliferation (two left panels) and induces apoptosis (two right panels), but also potentiates BRAFi activity (Fig. 2a). On the opposite, enforced expression of the two upregulated miRNAs (UPMIRNAs) miR-4443 and miR-4488 decreases the growth inhibitory effect of a BRAFi on cell viability (Fig. 2b, two left panels) and induction of apoptosis (Fig. 2b, two right panels). As expected, the simple overexpression of the two UPMIRNAs has no effect on melanoma cell growth. Furthermore, inhibiting UPMIRNAs expression by transient transfection of their respective antagomiRs in both drug sensitive and resistant M14 cells strongly impaired in vitro short-term colony formation (Fig. 2c). Likewise, overexpression of both DOWNMIRNAs miR-204-5p and miR-199b-5p strongly impaired colony formation in BRAFi-resistant cells (Fig. 2d).

**Fig. 3:**
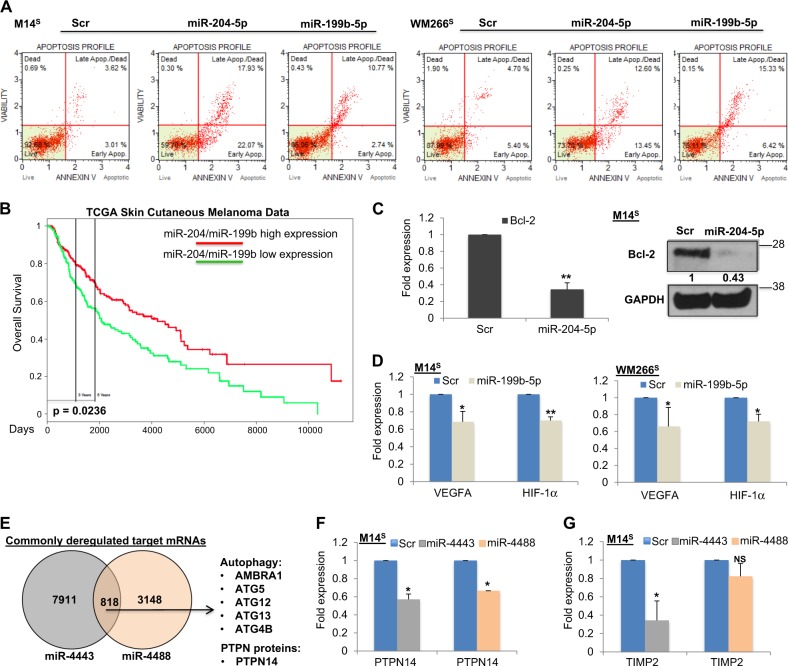
Shared deregulated miRNAs of drug-resistant melanoma cells act on cell intrinsic pathways. **a** FACS analysis showing that the overexpression of both miR-204-5p and miR-199b-5p induce M14S and WM266S cell apoptosis. **b** TCGA Skin Cutaneous Melanoma data revealing high co-expression of miR-204-5p/miR-199b-5p associated with a better survival probability (*p* = 0.0236). **c** Transient overexpression of miR-204-5p in M14S cells affecting Bcl-2 expression at mRNA (left panel) and protein (right panel) levels. **d** Enforced expression of miR-199b-5p reducing VEGFA and HIF-1α expression levels in M14S (left panel) and WM266S (right panel). **e** Venn Diagrams revealing the commonly deregulated target mRNAs of miR-4443 and miR-4488. **f** miR-4443 and miR-4488 overexpression reducing PTPN14 mRNA in M14S cells. **g** miR-4443 but not miR-4488 overexpression affecting TIMP2 mRNA in M14S cells

We then assessed the biological effects of the four selected miRNAs on cell intrinsic pathways. Starting from the two DOWNMIRNAs, we focused on their pro-apoptotic action on melanoma cell behavior through Caspase 3/7 activation. Results confirmed that the enforced expression of both miR-204-5p and miR-199b-5p induced melanoma cell apoptosis (Fig. 3a). Coherently with their emerging oncosuppressive role in melanoma, interrogating TCGA Skin Cutaneous Melanoma data (*n* = 501) we found that miR-204-5p and miR-199b-5p co-expression at high levels was associated with a better survival probability (*p* = 0.0236) for melanoma patients (Fig. 3b). No available TCGA data were found for miR-4443 and miR-4488.

miR-204-5p has been previously described as a negative regulator of Bcl-2 [24]. Indeed miR-204-5p enforced expression in M14^S^ melanoma cells reduced Bcl-2 expression levels both at RNA and protein levels (Fig. 3c, left and right panels). These results were confirmed also in WM266^S^ cells (Supplementary Fig. S3A). In addition, in line with previous reports we observed that enforced miR-204-5p expression was also able to inhibit the expression levels of RAD51, FOXM1 and NOTCH1 [18] (Supplementary Fig. S3B) [25–27]. Moreover, MAPKi-resistant melanoma cells consistently underwent a prominent increase of endogenous NOTCH1 levels and a cell-type dependent increase of the other two miR-204-5p targets RAD51 and FOXM1 (Supplementary Fig. S3C and D).

It has been reported that miR-199 family members are able to down-regulate the proliferative and pro-angiogenic HIF-1α/VEGF pathway [28]. Again, we confirmed that enforced expression of miR-199b-5p leads to downregulation of these targets both in M14 and WM266 (Fig. 3d). Finally, in long-term clonogenic assays with M14 drug sensitive cells [16] co-treatment with miR-199b-5p and a BRAFi for 28 days led to complete inhibition of clone formation as opposed to single BRAFi exposure, which only caused transient inhibition followed by appearance of resistant clones at later times (Supplementary Fig. S3E)

Finally, we started to investigate the potential mechanism of action of the two selected UPMIRNAs since little is known about miR-4443 and miR-4488 involvement in human cancers and in particular in melanoma. We conducted a bioinformatic analysis to identify the putative target genes of these microRNAs using several prediction tools [15] (for the complete list see Supplementary Table S1E and F). Among them, we found several autophagy-related genes such as *AMBRA1*, *ATG5*, *ATG12*, *ATG13* and *ATG4B* (Fig. 3e), which could be potentially downregulated in BRAFi-resistant melanoma cells. Interestingly, modulation of autophagy has been postulated to be a potential mechanism of adaptive resistance to MAPKi in sensitive and resistant BRAF-mutated melanoma cells [29]. A deeper assessment of miR-4443 and miR-4488 upregulation in the control of autophagy in drug-resistant cells is under investigation (L.F. and C.F.R. unpublished data).

Furthermore, among the predicted molecular targets of the UPMIRNAS we also found several protein tyrosine phosphatases (PTPs) known to antagonize the oncogenic activities of protein tyrosine kinases (TKs) (Supplementary Table S1E and F) [30]. In this context, PTPN14 was in common between the two (Fig. 3e). Therefore, we decided to validate this prediction through qRT-PCR following UPMIRNAs enforced expression in M14^S^ melanoma cells. Results (Fig. 3f) show that both miR-4443 and miR-4488 were able to inhibit the expression of this tumor suppressor phosphatase. Finally, miR-4443 was also reported to target the tissue inhibitor of metalloproteinase 2 (TIMP2) [31] which is involved in Vemurafenib resistance increasing melanoma invasiveness [32]. Hence, we assessed TIMP2 expression levels upon miR-4443 overexpression in M14^S^ melanoma cells. Results (Fig. 3g) confirmed this targeting. In contrast, as expected, miR-4488 was not able to inhibit TIMP2 expression levels. In line with these findings, MAPKi-resistant melanoma cells showed reduced expression levels of both TIMP2 and PTPN14 albeit in a cell-type-dependent manner (Supplementary Fig. S3F).

### Simultaneous co-targeting of deregulated miRNAs identified by whole miRNAome analysis potently affects drug-resistant melanoma cell growth

**Fig. 4:**
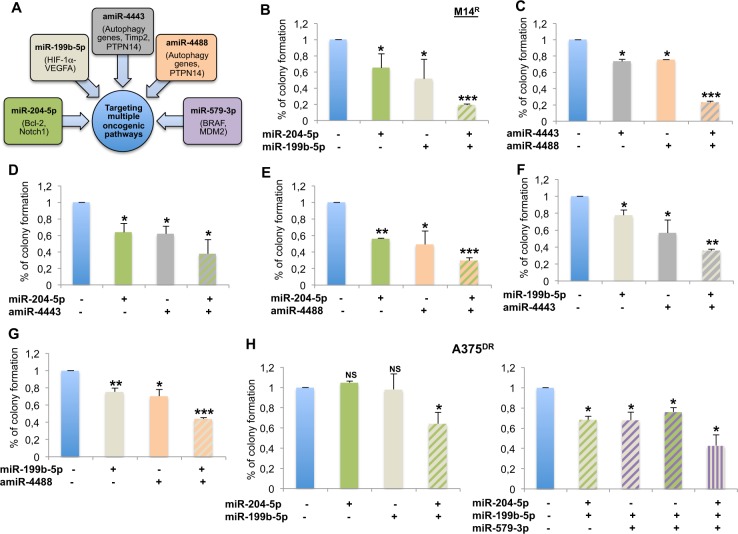
Simultaneous cotargeting of deregulated miRNAs affects drug-resistant melanoma cell growth. **a** Different miRNA action on non-redundant pathways relevant for melanoma progression. **b**–**g** miR-204-5p + miR199b-5p (**b**), amiR-4443 + amiR-4488 (**c**), miR-204-5p + amiR-4443 (**d**), miR-204-5p + amiR-4488 (**e**), miR-199b-5p + amiR-4443 (**f**) and miR-199b-5p + amiR-4488 (**g**) strongly reducing M14R cell colony formation as compared to single treatments. **h** Co-expression of miR-204-5p and miR-199b-5p mimics affecting A375DR cell colony formation as compared to single treatments (left panel) and even further when combined with miR-579-3p (right panel)

In line with the clinical need to develop new combination therapies capable to mitigate or block the development of drug resistance, we decided to assess the growth inhibitory effect of simultaneously targeting more than one deregulated miRNAs. This idea was supported by the prediction that the four selected miRNAs are postulated to act on non-redundant pathways relevant for melanoma progression as indicated in Fig. 4a. To this purpose, we tested the combinatorial treatments of different DOWNMIRNA mimics together with different UPMIRNA antagonists on MAPKi-resistant melanoma cell growth. All the combinations tested were able to strongly reduce M14^R^ melanoma cell colony formation as compared to single treatments: miR-204-5p + miR199b-5p, amiR-4443 + amiR-4488, miR-204-5p + amiR-4443, miR-204-5p + amiR-4488, miR-199b-5p + amiR-4443 and miR-199b-5p + amiR-4488 (Fig. 4b–g). These findings were confirmed in two additional melanoma cell lines rendered resistant to a BRAFi, namely WM266^R^ and A375^R^ (Supplementary Fig. S4A and B).

The effect of targeting miRNAs individually or in combination was assessed in a cell line rendered double resistant to both BRAF and MEK inhibitors (called A375^DR^). Others have previously reported that double-drug-resistant melanoma cell lines are more difficult to growth inhibit [6]. Indeed, we observed no growth inhibition effect on A375^DR^ when the four miRNAs were targeted individually (Supplementary Fig. S4C). In contrast, co-delivery of the two DOWNMIRNAs mimics resulted in a moderate inhibition of A375^DR^ melanoma cell growth (36%, *p* = 0.042) (Fig. 4h, left panel). When we combined them with the previously identified oncosuppressor miR-579-3p [16] a further enhancement of inhibition of A375^DR^ cell growth was observed compared to double treatments (from 32 to 58%, *p* = 0.0168) (Fig. 4h, right panel). These data suggest that the optimal approach to growth inhibit double-drug-resistant melanoma consists in the simultaneous co-targeting of multiple microRNAs.

### Drug resistant melanoma cells overproduce a wide array of proinflammatory and pro-angiogenic factors

**Fig. 5:**
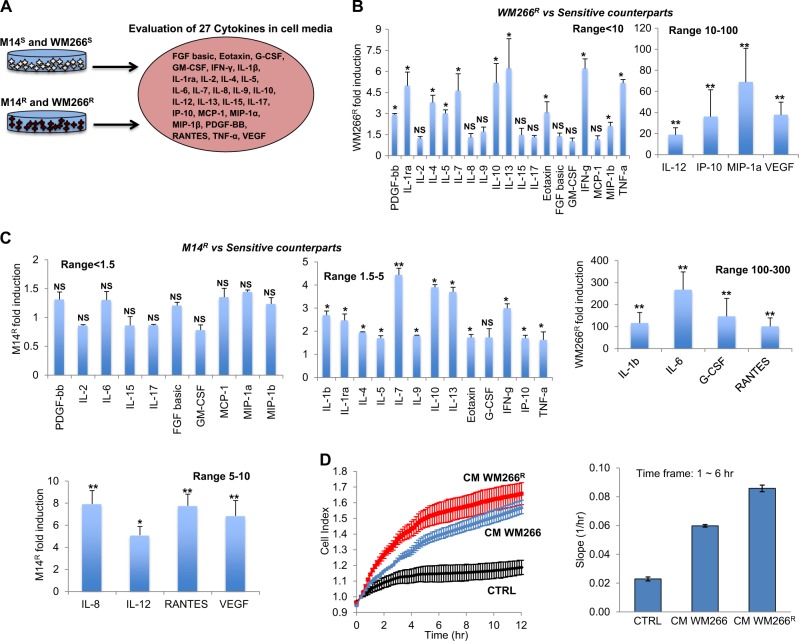
Conditioned media from drug-resistant melanoma cells is enriched in several proinflammatory and pro-angiogenic factors. **a** The levels of 27 cytokines determined in the supernatants from M14S and WM266SS cells and from their resistant counterparts. **b**, **c** WM266R and M14R cells conditioned media overproducing several cytokines and chemokines (fold change significance greater than 1.3) divided in three distinct groups with a high, medium and low degree of upregulation. **d** Cell index (left panel) and Slope induction (right panel) results obtained through xCELLigence Real Time Cell Analysis showing that conditioned media from WM266R melanoma cells inducing cell migration as compared to cell media from sensitive counterparts and CTRL media

As reported above, bioinformatics analysis of the predicted molecular targets of the commonly deregulated miRNAs in BRAF inhibitor-resistant cells highlighted a prominent involvement of pro-angiogenic and proinflammatory pathways. In order to validate these predictions, we decided to compare the cytokinome profile of drug resistant vs. drug sensitive WM266 and M14 melanoma cells. To this purpose, the levels of 27 cytokines were determined in cell-derived supernatants (Fig. 5a). As shown in Fig. 5b, c, both in M14 and, more pronounced (up to several-hundred folds) in WM266, a statistically significant (fold change significance greater than 1.3) increased secretion of a wide range of cytokines and chemokines was observed in drug resistant vs. drug sensitive cells. For each cell line, we could divide upregulated cytokines and chemokines in three distinct groups with a high, medium and low degree of upregulation, respectively.

Since several upregulated chemokines, cytokines and growth factors are involved in cell migration and metastasis we determined the capability of cell media from drug sensitive vs. resistant WM266 to elicit melanoma cell migration through xCELLigence Real Time Cell Analysis [21]. Results, expressed as Cell Index and Slope induction, clearly show that conditioned media from WM266^R^ melanoma cells strongly induced cell migration as compared to cell media from sensitive counterparts and CTRL media (Fig. 5d). These data were confirmed through canonical Boyden Chamber experiments (Supplementary Fig. S4D). Finally, upregulation of these proinflammatory and pro-angiogenic factors was confirmed at mRNA level in drug-resistant melanoma cells (Supplementary Fig. S5A). Of note, also in this case upregulation of cytokine genes was more pronounced in WM266 BRAFi-resistant cells. Cytokine perturbations in drug-resistant cells was confirmed also in A375^R^, LOX IMVI^R^ and A375^DR^ cells. In conclusion, BRAF resistant cells overproduce and release a wide spectrum of proinflammatory and pro-angiogenic factors and this is in line with what previously predicted from the alterations of the relevant microRNAs involved in controlling their expression.

### Down-modulation of miR-199b-5p in drug-resistant melanoma cells causes increased VEGF release and acquisition of a pro-angiogenic status

**Fig. 6:**
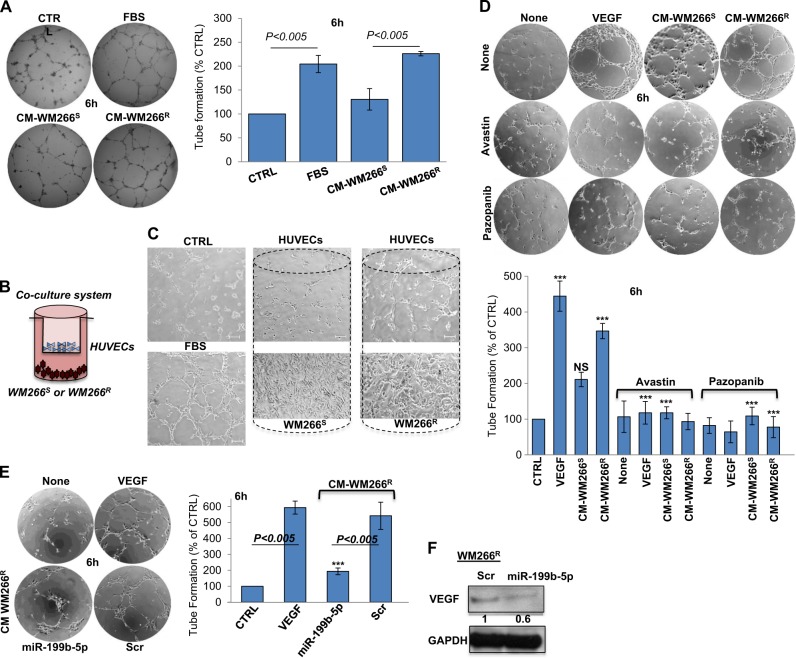
WM266R cells sustain pro-angiogenic stimuli through VEGFA release caused by downregulation of the oncosuppressive miR-199b-5p. **a** Conditioned media (CM) of drug sensitive vs. drug resistant WM266 cells inducing endothelial tube formation in human umbilical vein endothelial cells (HUVEC) plated on matrigel. **b** Non-contact co-culture system in which sensitive or BRAFi-resistant WM266 cells grown separated from HUVECs plated on matrigel in an intercup chamber exposed to their secretion products for 4 h. **c** Enhancing of cord-like structures of HUVECs co-cultured with WM266R cells as compared to HUVECs co-cultured with parental WM266S cells. Scale bar 100 μm. **d** Avastin (25 μg) or Pazopanib (5μg) inhibing tube formation induced by CM from WM266R melanoma cells; VEGF (200ng/ml) used as positive control. **e** miR-199b-5p enforced expression reducing the capability to induce tube formation of CM-WM266R cells. **f** Overexpression of miR-199b-5p inhibiting VEGF protein in WM266R cells observed through Western Blot analysis

VEGF was one of the most upregulated factors intercepted by cytokinome analysis of drug-resistant melanoma cells. This finding was of particular interest in the light of the known involvement of VEGF in melanoma progression [33]. Hence we decided to test the pro-angiogenic potential of the conditioned media (CM) of drug sensitive vs. drug resistant WM266 cells in inducing endothelial tube formation in human umbilical vein endothelial cells (HUVEC) plated on matrigel [17]. CM from WM266^R^ triggered already after 3 h (Supplementary Fig. S5B), a strong level of endothelial tube formation, whereas conditioned medium from WM266^S^ was inactive (Fig. 6a). As control, 10% FBS employed as a source of angiogenic growth factors, elicited a considerable response (Fig. 6a and Supplementary Fig. S5B). These data were confirmed through a non-contact co-culture system (Fig. 6b). Accordingly, tubes formed by cord-like structures in HUVEC co-cultured with WM266^R^ cells were strongly enhanced as compared to HUVEC co-cultured with parental WM266^S^ cells (Fig. 6c). To further confirm VEGF involvement, we specifically inhibited angiogenesis with Avastin or Pazopanib [34]. Results, reported in Fig. 6d and Supplementary Fig. S5C, confirmed that tube formation induced by CM from WM266^R^ melanoma cells was as efficient as recombinant VEGF and was significantly inhibited by the addition of either drug.

In order to find a correlation between miRNAs deregulation and VEGF increased production, we focused our attention on members of miR-199 family since they were reported to control VEGF expression [28]. Of notice, one of the most downregulated miRNA emerging our analysis of drug-resistant cells was miR-199b-5p. Hence, we decided to overexpress miR-199b-5p or the scrambled control in WM266^R^ cells. In line with our hypothesis CM from cells transfected with this miRNA lost the capability to induce tube formation (Fig. 6e). Finally, in order to assess whether miR-199b-5p was able to reduce specifically VEGF expression, we performed Western Blot analysis. Results (Fig. 6f) clearly showed that this was indeed the case.

All together, these findings support the notion that BRAFi-resistant melanoma cells are able to sustain pro-angiogenic stimuli through the increased release of VEGF, and that this is caused by downregulation of the oncosuppressive miR-199b-5p.

### Specific miRNAs signatures in tumour biopsies and blood characterize the acquisition of MAPKi-resistance in BRAF-mutated melanoma patients

**Fig. 7:**
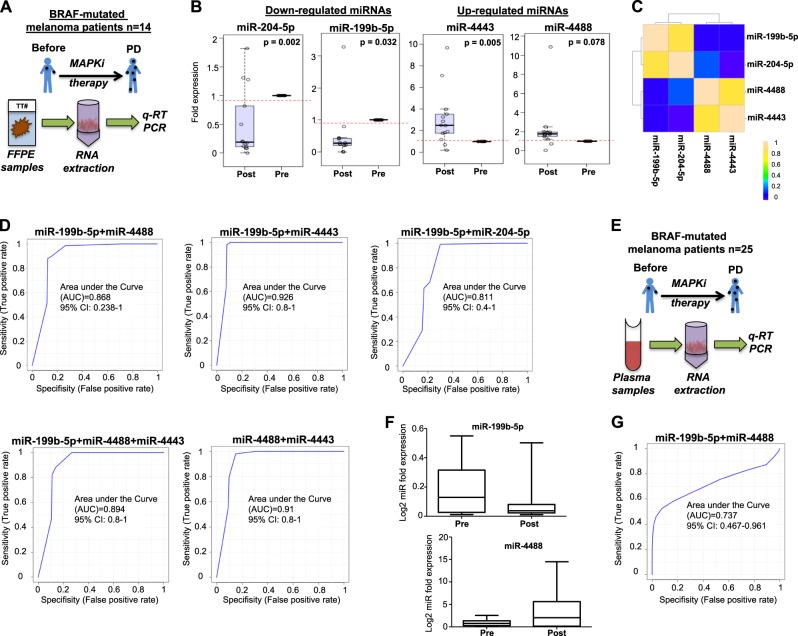
miRNAs signatures characterize the acquisition of MAPKi-resistance in BRAF-mutated melanoma patients. **a** Total RNA extracted from FFPE samples from 14 matched tumour samples before initiation of targeted therapy and after disease progression PD. **b** qRT-PCR results as box-whisker plots showing miR-204-5p/miR-199b-5p downregulation and miR-4443/miR-4488 upregulation in relapsing tumours. **c** Heat-map indicating the correlation of miR-199b-5p and miR-204-5p and anti-correlation to miR-4443 and miR-4488 (identified by yellow squares and by dark blue squares). **d** Receiver operating characteristic (ROC) curves estimating the predictive value of miRNA cormbinations (from FFPE samples) as markers of drug resistance. **e** Circulating microRNA extracted from the plasma of 25 melanoma patients before the beginning of therapy and at disease progression. **f** miR-199b-5p downregulation (higher panel) in the plasma of melanoma patients post-MAPKi treatment as compared to the plasma from untreated patients (pW = 0.17; pt = 0.19); miR-4488 upregulation (lower panel) in patients after MAPKi treatment (pW = 0.023; pt = 0.0039). **g** ROC curves estimating the predictive value of miR-199b-5p + miR-4488 cormbination as marker of drug resistance (from plasma samples). pt Student’s *t* test, pW Wilcoxon matched pairs test; pW = Wilcoxon matched pairs test. G, ROC curves estimating the predictive value of miR-199b-5p+miR-4488 cormbination as marker of drug resistance (from plasma samples)

The observations above suggest that changes in the level of expression of selected miRNAs could allow distinguishing BRAF-mutated melanomas after development of resistance to inhibitors of the MAPK pathway. Since miRNAs are very stable in formalin-fixed paraffin-embedded (FFPE) samples [16] total RNA was extracted from 14 matched tumour samples (before initiation of targeted therapy and after disease progression, PD) and subjected to qRT-PCR to determine the expression levels of mir-4443, miR-4488, miR-204b-5p and miR-199b-5p (Fig. 7a). Patients were all treated at therapeutic doses with the single-agent BRAF inhibitors vemurafenib (*n* = 10) or dabrafenib (*n* = 3) or with the combination of the BRAF inhibitor LGX818 and the MEK inhibitor MEK162 (*n* = 1). The clinical characteristics of the 14 advanced BRAF mutant melanoma patients included in our analysis are presented in Supplementary Table S2A. Results, shown as box-whisker plots, confirm in tumour samples previous results obtained in drug resistant vs. sensitive cell lines, i.e. that miR-204-5p and miR-199b-5p were strongly downregulated in relapsing tumours, whereas miR-4443 and miR-4488 were upregulated, albeit the second one not significantly (Fig. 7b). qRT-PCR results are also shown as box-whisker plots relative to each melanoma patient in Supplementary Fig. S6A. Moreover, the correlation index of the two-downregulated and the two upregulated miRNAs as a heat-map was assessed, through the measure of Pearson correlation coefficients. miR-199b-5p and miR-204-5p were found to be correlated with each other and anti-correlated to upregulated miRNAs, respectively (identified by yellow squares and by dark blue squares respectively in Fig. 7c). In contrast, miR-4443 and miR-4488 were found to have the opposite correlation.

A challenging issue in the therapy of cancer is the development of powerful tools able to predict patients' response to drugs. In this context, miRNAs could represent suitable candidates for the development of a non-invasive and reproducible predictive tool for their great stability [35]. Hence, the potential of the four identified up-or downregulated miRNAs was assessed. Their expression levels before therapy and after tumour progression were used to construct receiver operating characteristic (ROC) curves in order to estimate the predictive value of their deregulation as a marker of drug resistance. Sensitivity, specificity and accuracy of classifier was evaluated together by means of the Area Under Curve (AUC). Of importance, the two downregulated miRNAs, miR-199b-5p and miR-204-5p, yielded an area under the curve (AUC) of 0.79 and 0.64, with sensitivity reaching 100% (Supplementary Fig. S6B, two left panels). On the other hand, the two upregulated miRNAs, miR-4488 and miR-4443, yielded an AUC of 0.85 with sensitivity reaching 100% (Supplementary Fig. S6B, two right panels). Thereafter, the correlation between changes in the expression of combinations of miRNAs between pre- and post-treatment samples was measured (Fig. 7d). Again, ROC curves were plotted for the best combinations of the four miRNAs and a 95% of power at a significance level of 0.05 was considered to detect a value of AUC of 0.75 as significant with respect to the null hypothesis value of 0.50. Interestingly, as shown in Fig. 7d significant AUC values were obtained for several combinations: miR-199b-5p + miR4488, miR-199b-5p + miR-4443, miR-199b-5p + miR-204-5p, miR-199b-5p + miR-4443 + miR-4488 and miR-4443 + miR-4488. The highest AUC values of 0.926 and 0.91 were observed in the case of miR-199b-5p + miR-4443 and miR-4488 + miR-4443, respectively. All together these data strongly support the concept that the measurement of miRNAs deregulation could allow to develop powerful predictive tools to identify patients who develop drug resistance in metastatic melanoma.

Finally, the levels of expression of miR-199b-5p and miR-4488 were determined in the plasma of 25 melanoma patients before the beginning of therapy and at disease progression (Fig. 7e). Patients were all treated at therapeutic doses with the single-agent BRAF inhibitors vemurafenib (*n* = 9), with the combination of the BRAF inhibitor LGX818 and the MEK inhibitor MEK162 (*n* = 7) or with the combination of the BRAF inhibitor vemurafenib and the MEK inhibitor cobimetinib (*n* = 9). The clinical characteristics of the 25 advanced BRAF-mutant melanoma patients included in our analysis are in Supplementary Table S2B. We evaluated miR-199b-5p since miR-199 family members are widely describe to be potential human biomarkers [36, 37] whereas miR-4488 has been previous described to be a circulating microRNA for early breast cancer detection [38]. Coherently with the previous findings, miR-199b-5p expression levels were downregulated in the plasma of melanoma patients post-MAPKi treatment as compared to the plasma from untreated patients (Fig. 7f, upper panel). Again, miR-4488 levels were significantly increased in patients after MAPKi treatment (Fig. 7f, lower panel). Of note these data were normalized using two different methods: global mean normalization (GMN) and NormFinder model [19], which gave very similar results (Supplementary Table S2C) confirming the magnitude and the directionality of our analysis. The expression levels of these two miRNA were used to plot ROC curves for single miRNAs (Supplementary Fig. S6C) and in combination. This resulted in a significant AUC value of 0.737 (Fig. 7g) coherently to what observed in tumor samples (Fig. 7d, first panel). These findings suggest the possibility that the simultaneous assessment of deregulated miRNAs in human samples could represent a valuable tool to identify melanoma patients sensitive vs. resistant to therapy with MAPK inhibitors.

## Discussion

The mechanisms at the basis of drug resistance are under intense investigation. Resistance has been classically recognized as the product of secondary mutations. However, in most cases there is no identifiable genetic cause. In line with this, in recent years, a growing number of studies in metastatic melanoma have pointed to the role of non-genetic mechanisms as responsible for the emergence of resistant populations of cancer cells under selective drug pressure [39]. Among most, well-studied non-genetic mechanisms of resistance in melanoma are as follows: (a) autocrine activation of receptor tyrosine kinases such as ERBB3 which drives pAKT-dependent cell survival and proliferation [10, 11]; (b) enhanced mitochondrial biogenesis and aberrant bioenergetics, which makes the tumor increasingly dependent upon oxidative phosphorylation and HSP90 activity[7]; (c) drug induced changes in the immune and inflammatory niches that increase drug tolerance to MAPK inhibitors [22, 40]. In this context it has to be highlighted the recent identification of transcriptional variability acting at a single cell level and capable to predict which cells are capable to resist drug treatment, and of epigenetic reprogramming events responsible for the conversion of transient transcriptional states into stably resistant ones [41]. Likewise a thorough study of the dynamic transcriptomic changes occurring progressively both in in vitro cells lines exposed to MAPKi and in *on-treatment* patient samples has allowed to delineate the development of transcriptomic trajectories which accompany the evolution of drug resistance [13]. However, so far, no study has addressed in detail the involvement of post-transcriptional events and in particular how global changes in the expression of the most pleiotropic post-transcriptional regulators, namely microRNAs, could coordinate the development of drug resistance. This was the object of the present study.

We have analyzed the temporal evolution of global miRNAome changes in two BRAF-mutated melanoma cells exposed to growing drug concentrations, have derived shared patterns of miRNA changes and have confirmed several of these changes in additional cell lines and in biopsies of patients, which underwent tumor progression upon MAPKi therapy. Several are the lessons learned. Firstly, we observed an increasing number of commonly deregulated miRNAs which approached at the highest drug concentration 10% of the total number of miRNA analyzed. Furthermore, we showed that the global expression pattern of miRNA expression was capable of distinguishing cell populations with different degrees of drug resistance. Secondly, deregulated miRNA collectively control a growing number of signaling pathways which affect both cell intrinsic growth behaviors such as resistance to apoptosis and autophagy, as well as drivers of the interactions of melanoma cells with the tumor microenvironment through the increased secretion of a large set of proinflammatory and pro-angiogenic factors. Taken together these observations lead us to conclude that global miRNA expression changes in drug-resistant cells recapitulate and are the mirror image of what has previously been observed at mRNA transcriptomic level: i.e. a switch to a cytokine signaling/migration and extracellular matrix remodeling/angiogenesis condition [13]. It is as if melanoma cells during their gradual road to resistance undergo not only a mRNA global transcriptomic reprogramming but also a parallel miRNA reprogramming, in order to consolidate and stabilize their drug-resistant status.

The present study has analyzed in detail the behavior and biological impact of a subset of miRNAs, two of which downregulated during progression to drug resistance (DOWNMIRNAs), the other two which undergo upregulation (UPMIRNAs). While the two DOWNMIRNAs had already been reported to be involved in melanoma development [42, 43] but not in drug resistance, for the two UPMIRNAs their involvement in melanoma as oncomiRs is novel. The role of the two DOWNMIRNAs as oncosuppressors was supported by the evidence that high levels of co-expression of these two miRNAs correlate with increase survival. This was further confirmed by the demonstration that among major targets of miR-204 were pro-tumorigenic factors such as Bcl-2, FOXM1, Notch and RAD51 and, in the case of miR-199, VEGF production. In this last case we showed convincingly that the pro-angiogenic properties of drug-resistant melanoma cells rely on the disregulation of a miR-199/VEGF axis which could be reversed by reinstalling high cellular levels of miR-199. Much less was known about the two UPMIRNAs miR-4488 and miR-4443. In this case highly intriguing was our observation of the enrichment of autophagy proteins (AMBRA1, ATG5, ATG12, ATG13 and ATG4B) among their postulated targets. These effectors attend to different stages of the autophagic process from induction (ATG13) to nucleation (AMBRA1), to elongation (ATG5, ATG12 and ATG4B) leading to the formation of the double-membrane autophagosome [44]. During last years the role of autophagy is emerging in anticancer therapy and also in the context of MAPKi inhibitors in BRAF-mutated metastatic melanoma [45]. In this context, heterozygous deletion of Atg5 in genetically engineered mice enhanced melanoma metastasis and worsened the response to dabrafenib [46]. Hence, one can postulate that deregulation of autophagy could represent a protective mechanism to contrast the growth inhibitory effect of BRAF inhibition following UPMIRNAs overexpression in melanoma cells. A deeper assessment of the role of miR-4443 and miR-4488 upregulation in the control of autophagy in drug-resistant cells is currently under investigation (L.F. and C.F.R. unpublished).

Among the predicted molecular targets of the UPMIRNAs, we found several protein tyrosine phosphatases (PTPs) whose loss has been shown to promote tumorigenesis in different in vitro and in vivo models [30]. In particular, PTPN14, a molecular target shared by miR-4443 and miR-4488, has been described to attenuate the oncogenic function of the Hippo pathway effector YAP1 [47]. In this context, activation of YAP/TAZ is emerging as a mechanism of melanoma resistance to MAPK inhibitors [48]. BRAF inhibitors increase melanoma cell contractility, induce changes in cell shape by rewiring their cytoskeleton and activating the YAP/TAZ mechanotransduction pathway [49]. In line with this, it is interesting to highlight that also the tissue inhibitor of metalloproteinase 2 (TIMP2), which is involved in vemurafenib-induced collagen disorganization and extracellular matrix alteration, is a molecular target of the UPMIRNA miR-4443 [32]. These findings together pave the way to study the role of miRNAs in the remodelling of cell cytoskeleton and in the activation of YAP/TAZ mechanotransduction pathway in melanoma drug resistance. Likewise, it will be important to further dissect if miRNA deregulation is contributing to modify the reciprocal interaction of melanoma cells with other cells of the tumour microenvironment such as CAFs, macrophages, NK cells and cells of the adaptive immune system.

Our observations have important therapeutic implications for the control of drug resistance in melanoma. Reverting the expression of deregulated miRNAs by either enforcing expression of DOWNMIRNAs or inhibiting expression of UPMIRNAs was able consistently to impair in concert with BRAF and MEK inhibitors the emergence of drug resistance in vitro in short term as well as in long-term clonogenic assays. The most intriguing conclusion derives from the analysis of drug-resistant melanoma cells. Here, a single DOWNMIRNA was unable to affect cell growth, an effect that was obtained instead when two or, even three different DOWNMIRNAs were administered. The most likely explanation is that double drug-resistant melanoma cells activate, in order to survive, a plethora of parallel oncogenic pathways, which require in order to be rewired their simultaneous inhibition by a combined set of pleiotropic inhibitors. This observation has to be taken into account in the development of novel combination therapies in particular those based on the nanodelivery of miRNA-carrying particles as they have the required flexibility to carry different miRNA oncosuppressor cargos [50].

Finally, our data using matched tumour biopsies and serum samples pre-treatment and after tumour progression highlight the possibility to derive simple miRNA signatures capable potentially to distinguish drug responding from non responding patients. Indeed, using only two deregulated miRNAs as biomarkers was it possible to discriminate pre-treatment from tumour progression samples with the desired sensitivity and specificity. These data, obtained in retrospective cohorts of patients need to be confirmed and expanded in prospective statistically powered studies, perhaps by including additional deregulated miRNAs. Nevertheless, the robust set of data obtained so far, encourage us to pursue this avenue in order to develop circulating miRNA-based non invasive predictive tools as a non-invasive measure of drug sensitivity or resistance.

## Electronic supplementary material

Suppl. Figure 1

Suppl. Figure 2

Suppl. Figure 3

Suppl. Figure 4

Suppl. Figure 5

Suppl. Figure 6

Suppl. Table 1

Suppl. Table 2

Suppl. Figure Legends
