# Supplementary figures and images for: Reprogramming miRNAs global expression orchestrates development of drug resistance in BRAF mutated melanoma
Source: Cell Death Differ. 2018 Sep 25;26(7):1267–82. doi: 10.1038/s41418-018-0205-5 (PMC6748102; doi:10.1038/s41418-018-0205-5)

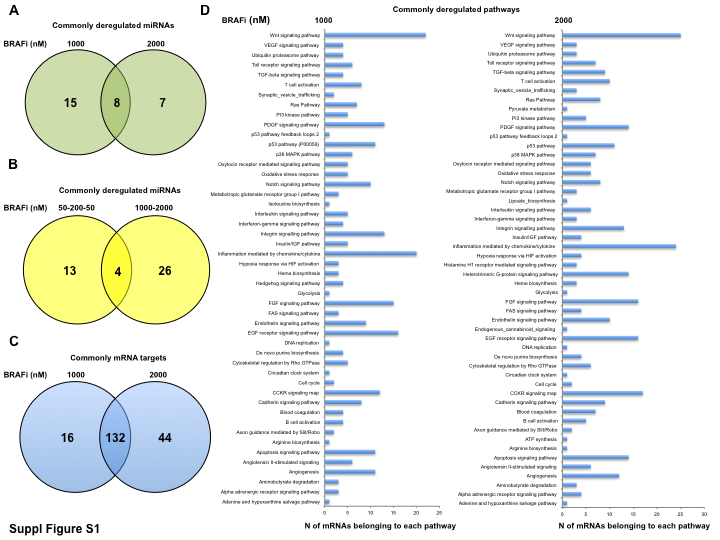

Supplement: Supplementary file 1 — Suppl. Figure 1 [file 41418_2018_205_MOESM1_ESM.tif]

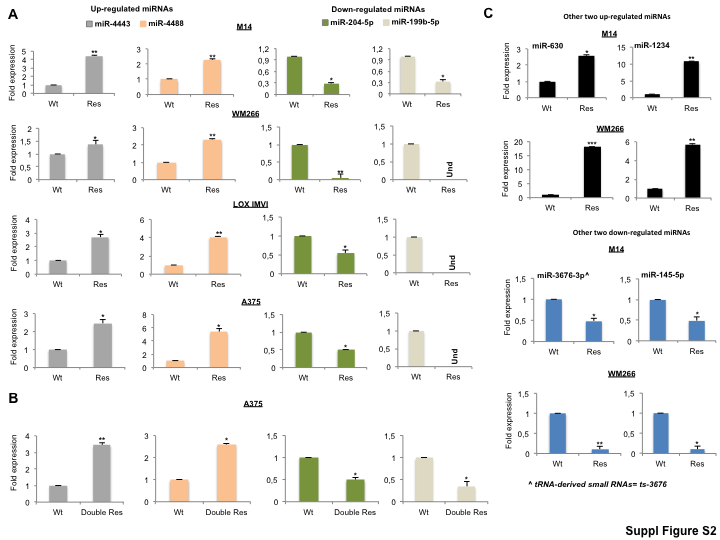

Supplement: Supplementary file 2 — Suppl. Figure 2 [file 41418_2018_205_MOESM2_ESM.tif]

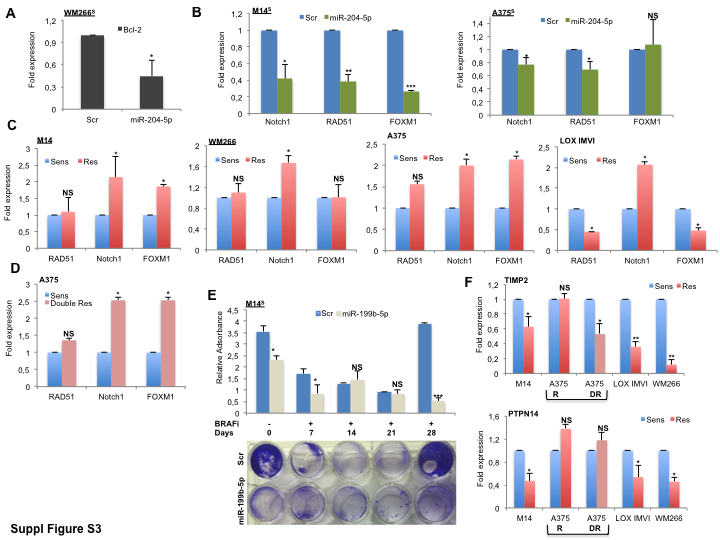

Supplement: Supplementary file 3 — Suppl. Figure 3 [file 41418_2018_205_MOESM3_ESM.tif]

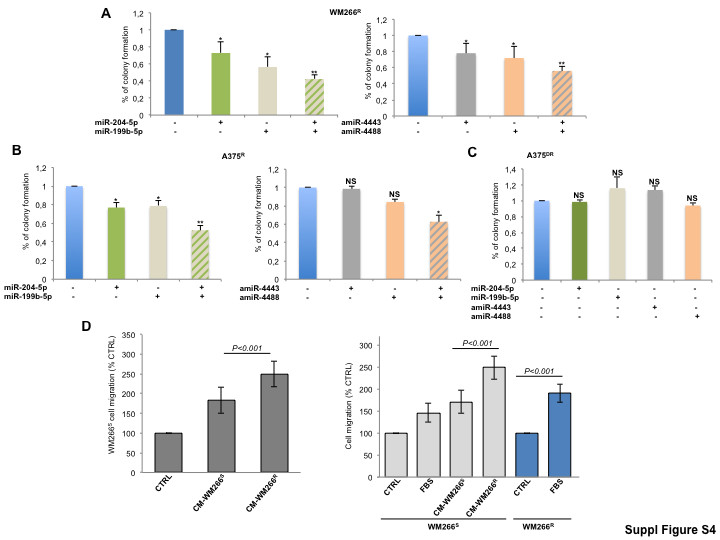

Supplement: Supplementary file 4 — Suppl. Figure 4 [file 41418_2018_205_MOESM4_ESM.tif]

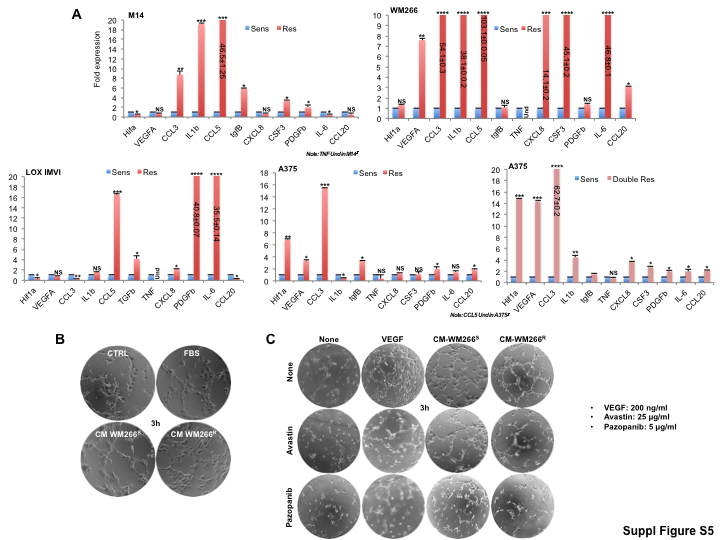

Supplement: Supplementary file 5 — Suppl. Figure 5 [file 41418_2018_205_MOESM5_ESM.tif]

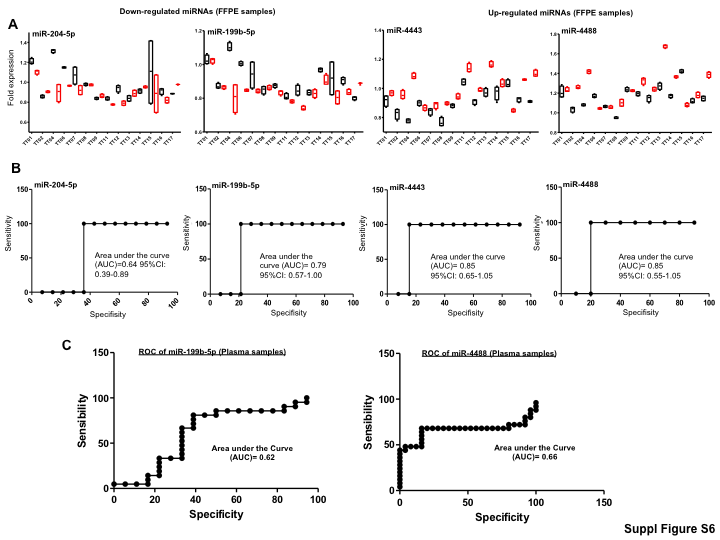

Supplement: Supplementary file 6 — Suppl. Figure 6 [file 41418_2018_205_MOESM6_ESM.tif]
