# Supplementary material for: Reprogramming miRNAs global expression orchestrates development of drug resistance in BRAF mutated melanoma
Source: Cell Death Differ. 2018 Sep 25;26(7):1267–82. doi: 10.1038/s41418-018-0205-5 (PMC6748102; doi:10.1038/s41418-018-0205-5)
Supplement: Supplementary file 9 — Suppl. Figure Legends [file 41418_2018_205_MOESM9_ESM.docx]

**SUPPLEMENTARY FIGURE AND TABLE LEGENDS**

**Supplementary Figure S1.** Commonly deregulated miRNAs at the higher doses of selection affect several intracellular pathways. **A,** Venn Diagram showing that the highest steps of selection (i.e. 1μM and 2μM BRAFi) corresponding to the greatest number of commonly deregulated miRNAs between the two cell lines. **B,** Lower steps of selection (i.e. 50-200-500 nM) sharing only a small number of deregulated miRNAs with the highest selection steps. **C,** Approaching 70% of the total predicted target mRNAs of commonly deregulated miRNAs shared between the 1 and 2 μM steps of selection. **D,** Bioinformatics analysis of the predicted molecular pathways of the commonly deregulated miRNAs at the highest doses of selection.

**Supplementary Figure S2.** Validation of Nanostring data by Real Time-PCR on a subset of deregulated miRNAs in several MAPKi-resistant melanoma cells. **A,** qRT-PCR results showing miR-4443/miR-4488 upregulation and miR-204-5p/miR-99b-5p downregulation in four melanoma cells rendered resistant to a BRAFi. **B,** The deregulation of these four miRNAs confirmed in a cell line rendered double resistant to both BRAF and MEK inhibitors (A375^DR^). **C,** Nanostring data validation of other three additional miRNAs (i.e. miR-630, miR-1234 and miR-145-5p) and of a tRNA-derived small RNAs (tsRNAs), namely ts-3676.

**Supplementary Figure S3.** The four deregulated miRNAs of drug resistant melanoma cells act on cell intrinsic pathways. **A,** Transient overexpression of miR-204-5p in WM266^S^ cells affecting Bcl-2 expression at RNA levels. **B,** miR-204-5p reducing Notch1, RAD51 and FOXM1 expression levels in M14^S^ and A375^S^ cells. **C, D,** MAPKi-resistant melanoma cells showing an increase of endogenous NOTCH1 levels and a cell-type dependent increase of the other two miR-204-5p targets RAD51 and FOXM1. **E,** Long-term clonogenic assays on M14^S^ demonstrating that co-treatment with miR-199b-5p and a BRAFi for 28 days leading to complete inhibition of clone formation. **F,** MAPKi-resistant melanoma cells showing reduced expression levels of both TIMP2 and PTPN14 albeit in a cell-type dependent manner.

**Supplementary Figure S4.** Therapeutic miRNA combinations affect drug resistant melanoma cell growth. **A, B,** miR-204-5p+miR199b-5p (left panels) and amiR-4443+amiR-4488 (right panels) reducing WM266^R^ and A375^R^ cell colony formation as compared to single treatments. **C,** The four miRNAs targeted individually having no growth inhibition effect on A375^DR^ cells. **D,** Boyden Chamber experiments showing that conditioned media from WM266^R^ melanoma cells induced cell migration.

**Supplementary Figure S5.** MAPKi-resistant melanoma cells up-regulation of several pro-inflammatory and pro-angiogenic factors at RNA levels. **A,** qRT-PCR results confirming the up-regulation of several pro-inflammatory and pro-angiogenic factors in different drug resistant melanoma cells. **B,** Conditioned media (CM) of drug sensitive vs drug resistant WM266 cells inducing endothelial tube formation in human umbilical vein endothelial cells (HUVEC) plated on matrigel already after 3 h. **C,** VEGF inhibition by Avastin (25μg) or Pazopanib (5μg) reducing tube formation induced by CM from WM266^R^ melanoma cells; VEGF (200ng/ml) used as positive control.

**Supplementary Figure S6.** miRNAs deregulation characterize the acquisition of MAPKi-resistance in BRAF-mutated melanoma patients. **A,** qRT-PCR results as box-whisker plots relative to each melanoma patient showing miR-204-5p/miR-199b-5p down-regulation and miR-4443/miR-4488 up-regulation in relapsing tumours. **B,** Receiver operating characteristic (ROC) curves estimating the predictive value of single miRNA (from FFPE samples) as markers of drug resistance. **C,** ROC curves valutating the predictive value of miR-199b-5p (left panel) and miR-4488 (right panel) as markers of drug resistance (from plasma samples).

**Supplementary Table S1.** Bioinformatics analysis on Nanostring data and predicted target mRNAs of deregulated miRNAs. **A, B,** Complete statistical analysis of Nanostring results on the different selection steps of M14 (**A**) and WM266 (**B)** cells. **C,** The entire list of statistically significant deregulated miRNAs at the highest doses of selection (i.e. 1μM and 2μM BRAFi). **D,** Lists of the 148 and 176 predicted mRNA targets of commonly deregulated miRNAs between the two cell lines at the doses of 1 and 2 μM. **E, F,** Bioinformatics analysis identifying the putative target genes of miR-4443 (**E**) and miR-4488 (**F**) using several prediction tools.

**Supplementary Table S2.** Clinical characteristics of the patients included in our analysis. **A,** FFPE samples derived from 14 BRAF-mutated melanoma patients treated with therapeutic doses of the single-agent BRAF inhibitor vemurafenib (n = 10) or dabrafenib (n = 3) or with the combination of the BRAF inhibitor LGX818 and the MEK inhibitor MEK162 (n = 1). **B,** Plasma samples derived from 25 BRAF-mutated melanoma patients treated at therapeutic doses with the single-agent BRAF inhibitors vemurafenib (n = 9), with the combination of the BRAF inhibitor LGX818 and the MEK inhibitor MEK162 (n = 7) or with the combination of the BRAF inhibitor vemurafenib and the MEK inhibitor cobimetinib (n= 9). **C,** Plasma samples derived results normalized using two different methods: global mean normalization (GMN) and NormFinder model.
